# Echocardiographic pattern of rheumatic valvular disease in a contemporary sub-Saharan African pediatric population: an audit of a major cardiac ultrasound unit in Yaounde, Cameroon

**DOI:** 10.1186/s12887-016-0584-z

**Published:** 2016-03-21

**Authors:** Clovis Nkoke, Alain Lekoubou, Anastase Dzudie, Ahmadou Musa Jingi, Samuel Kingue, Alain Menanga, Andre Pascal Kengne

**Affiliations:** Department of Internal Medicine and Medical Specialties, University of Yaounde 1, Yaounde, Cameroon; Department of Neurosciences, Division of Neurology, Medical University of South Carolina, Charleston, SC USA; Department of Internal Medicine, Douala General Hospital, Douala, Cameroon; Non-Communicable Diseases Research Unit, South African Medical Research Council, Cape Town, South Africa; Department of Medicine, Groote Schuur Hospital, University of Cape Town, Cape Town, South Africa; The George Institute for Global Health, University of Sydney, Sydney, Australia

**Keywords:** Rheumatic heart disease, Children, Cameroon

## Abstract

**Background:**

Rheumatic Heart Disease (RHD) remains a major cause of childhood acquired heart disease in developing countries. However reported echocardiographic features are limited to a few countries. This report is on the demographic and echocardiographic features of RHD in children using data from the largest referral hospital in Yaounde, the capital city of Cameroon.

**Methods:**

The register of the cardiac ultrasound unit of the Yaounde General Hospital for the period 2003–2013 served as basis for data collection. RHD diagnosis was based on the World Heart Federation Criteria for the diagnosis of RHD. Demographic data, pattern of valve lesions and severity were analyzed.

**Results:**

A total of 1130 first echocardiographic examinations were performed in children aged ≤ 18 years. Sixty-five (5.8 %) had a definite echocardiographic diagnosis of RHD with their mean age being 11.8 years (SD 3.6) and 31 (47.1 %) being boys. The commonest primary reasons for requesting an echocardiographic examination were a clinical diagnosis of RHD (24.6 %) without heart failure, a clinical diagnosis of heart failure (24.6 %), and heart murmurs (21.5 %). Isolated mitral regurgitation was the most common valve lesion (49.2 %) and was frequently associated with aortic regurgitation (35.4 %). Severe lesions were found in 63.3 % of participants. No right heart lesion was reported.

**Conclusions:**

A sizable proportion of children undergoing echocardiographic examination at this major referral hospital in Cameroon had RHD, with lesions found only on the left heart. These lesions predominated on the mitral valve, were commonly associated with aortic regurgitation, and more often severe.

## Background

Rheumatic heart disease (RHD) is the most common acquired heart disease in childhood. It remains a major public health issue in children and young adults in low and middle income countries [1–4]. It is the leading cause of heart failure in children and adults in Africa [1]. RHD results from repeated acute rheumatic fever attacks subsequent to Group A streptococcal throat infections. In Africa, reports on RHD in the pediatric population originate mainly from a few countries and not so many have described the echocardiographic patterns of rheumatic valve lesions [5–8]. Mitral valves are traditionally more affected, followed by aortic, tricuspid and pulmonic valves. It is unclear whether the same pattern applies to pediatric populations across all countries in sub-Saharan Africa (SSA) and whether there are some distinctive features for the SSA pediatric population that remains to be determined. We sought in this paper to describe the echocardiographic pattern of RHD in children using data from the largest cardiac echography center in Cameron, SSA.

## Methods

### Study setting

The Yaoundé General Hospital is a major cardiac referral hospital in the Capital city of Cameroon, with a catchment population of about 2 million inhabitants in 2011. The hospital receives patients referred from other health institutions nationwide for the investigation and/or management of suspected heart disease. At the end of 2013, the echocardiography unit was staffed with four cardiologists with expertise in echocardiography.

The echocardiography unit’s register was surveyed for the period 2003 through 2013 to identify patients with the diagnosis of RHD. Only the first echocardiographic examination report for each patient was included. Using a pre-defined questionnaire, we extracted data on age at the time of diagnosis, sex, clinical indication for the echocardiography, and the echocardiographic description of the four valves and diagnosis.

The diagnosis of RHD was based on the World Heart Federation (WHF) criteria for echocardiographic diagnosis of RHD. Briefly, RHD was defined by the presence of any evidence of mitral or aortic regurgitation seen in two planes associated with at least two of the following morphologic abnormalities of the regurgitating valve: restricted leaflet motility, focal or generalized valvular thickening, and abnormal sub-valvular thickening [9]. The echocardiographic reports were reviewed and used as the basis of RHD diagnosis.

Only those patients with sufficient information to make the diagnosis of RHD by the WHF criteria were included. Those with isolated functional regurgitating lesions were excluded from the analysis. Data were summarized by the type of valves involved, pathology (i.e., stenosis vs. regurgitation), and severity described as mild, moderate, and severe according to American College of Cardiology/American Heart Association (ACC/AHA) guidelines [10]. The study was approved by the administrative authorities of the hospital (Chief Executive Officer, CEO), in the absence of an institutional review board. The CEO granted permission to access and use the data from the registers for research purpose, in respect of the confidentiality of data archived in the registers. No informed consent was obtained from patients since it was a retrospective survey of the echocardiography register, with no contact between study investigators and individual patients. Furthermore, the authors are aware of no existing national research guidelines in the study setting.

### Statistical analysis

Data were analyzed with the use of IBM® SPSS® Statistics version 22 for Windows ®. We have presented the results as count and percentage for qualitative variables, and means and standard deviation for quantitative variables. Comparisons across groups used the chi square test, the t-student tests and equivalents as appropriate. A *p*-value <0.05 was considered statistically significant.

## Results

### Demographic characteristics and referral for echocardiography

**Fig. 1:**
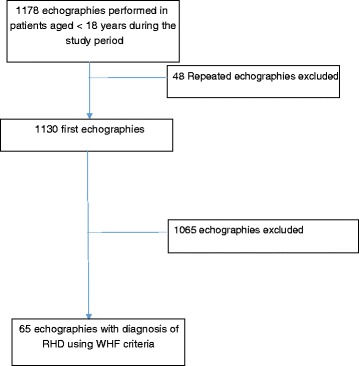
Flow chart for patients selection

A total of 65 participants out of 1130 (5.8 %) aged 18 year old or younger met the diagnostic criteria for RHD (Fig. 1). Out of the 65 children, 31 (47.7 %) were males. Participants’ age ranged from 4 to 18 years, with a mean of 11.8 (standard deviation 3.6) years. A clinical indication and reason for referral were available for 84.6 % of the children (55 of 65). The most common reasons for performing the echo was a clinical suspicion of RHD and a clinical diagnosis of heart failure with 16 patients (24 %) for each indication. Other indications included heart murmurs in 14 (21.5 %) children, dyspnea in 4 (6.2 %) patients, suspicion of congenital heart disease, palpitations, chest pain, cardiomegaly on chest X-ray, and atrial fibrillation in one patient each (1.5 %).

### Pattern of rheumatic valve disease

Isolated mitral valve regurgitation was the most common rheumatic lesion, found in 32 (49.2 %) children, followed by the combination of mitral and aortic valve regurgitation in 23 (35.4 %) children. Stenotic lesions were infrequently reported (between 1.5 % for isolated aortic stenosis and 6.2 % for a combination of mitral regurgitation and stenosis (Table 1). No lesion was detected on right heart valves. The tricuspid regurgitation reported in 6 (9.2 %) children was functional. The severity of valve lesions was available for 60 (92.3 %) children. Overall, 38 (63.3 %) of lesions were classified as severe, 19 (31.6 %) as moderate, and 3 (5 %) as mild. Mitral regurgitation was evenly distributed between boys and girls (16 children each), however the combination of mitral regurgitation and mitral stenosis was found only in boys (100 vs. 0 %, *p* = 0.046) and mitral and aortic regurgitations were more frequently seen in combination in girls (72.7 vs. 27.3 %, *p* = 0.018). No sex-difference was observed in the distribution of other valve lesions. No difference between boys and girls was observed for age in relation with isolated mitral valve regurgitation (mean age 12.4 ± 2.8 years in boys vs. 11.3 ± 3.6 years in girls, *p* = 0.748), isolated aortic regurgitation (11.7 ± 3.5 years in boys vs. 12 ± 3.7 years in girls, *p* = 0.737), or combined aortic and mitral regurgitations (11.9 ± 3.4 years in boys vs. 11.7 ± 4.0 years in girls, *p* = 0.814).

**Table 1:** Distribution of valvular lesions

Valvular lesions	Overall, *n* (%)	Boys, *n* (%)	Girls, *n* (%)
Mitral regurgitation only	32 (49.2)	16 (50)	16 (50)
Mitral stenosis only	1 (1.5)	0	1 (100 %)
Mitral regurgitation + mitral stenosis	4 (6.2)	4 (100)	0
Aortic regurgitation only	7 (10.8)	6 (85.7)	1 (14.3)
Aortic stenosis only	2 (3.1)	1 (50)	1 (50)
Aortic regurgitation + aortic stenosis	2 (3.1)	1 (50)	1 (50)
Mitral regurgitation + aortic regurgitation	23 (35.4)	6 (27.3)	16 (72.7)
Mitral regurgitation + aortic stenosis	1 (1.5)	0	1 (100)
Mitral regurgitation + mitral stenosis + aortic regurgitation	2 (3.1)	2 (3.1)	0
Mitral regurgitation + aortic regurgitation + aortic stenosis	1 (1.5)	0	1 (100 %)
Pulmonary hypertension	34 (52.3)	15 (44.1)	19 (55.9)
Overall	65 (100 %)	31 (47.7)	34 (52.3)

## Discussion

In this study covering about a decade of activities in a major referral echocardiography unit in Cameroon, we found that RHD in children represented about 6 % of all first echocardiographic examinations in the pediatric population. Heart failure and clinical suspicion of RHD were the most common indications or reasons for referral for echocardiography. The most common rheumatic valve lesion was mitral regurgitation, a pattern that was often associated with aortic regurgitation with the latter being more common among girls. No rheumatic lesion was found on the tricuspid and pulmonic valves.

An echocardiographic screening study in Uganda in 2010 showed that the prevalence of RHD among school children was 1.5 % [6]. A similar screening study in Mozambique in 2005 revealed a prevalence of 3 % [7]. The equivalent figure in Senegal was 0.75 % [8]. We found a higher prevalence which could be explained by the fact that our study was not a screening study with all our patients having either specific or non-specific cardiac symptoms. The THESUS-HF registry of 1006 patients with heart failure from 9 African countries showed that RHD was the third most important cause of heart failure in adult Africans [11]. Altogether our findings and those previously reported in SSA suggest that, as previously highlighted in their review by Carapetis et al. [2], SSA is currently the RHD ‘hot spot’ of the world. Data from other developing countries outside SSA shows a comparable prevalence. For instance, in India, Several reports on echocardiography screening of school-aged children have yielded a prevalence ranging from 0.1 to 5.1 % [12–17].

The distribution of rheumatic valve lesions in our study mirrors those in previous reports both from developed and developing countries where the order of involvement is mitral followed by aortic, tricuspid and pulmonic valves [3, 18]. Several studies have now almost unanimously highlighted the superiority of echocardiography over clinical examination for the diagnosis of clinical or subclinical RHD lesions [2, 5, 17, 19, 20]. This may translate into a widespread use of echocardiography in areas with prevalent RHD. However, limited financial resources and staff with echocardiography skills in developing countries are deterrents to such an approach. While cardiac ultrasound may become the gold standard in diagnosing RHD in resource-limited settings, caution should be exercised when interpreting data from Doppler echocardiography, especially if the findings are isolated and without any clinical evidence. Not less important, physiological regurgitation is not uncommon in children and has to be carefully excluded prior to any diagnosis of organic valvular lesion [21]. While for the purpose of comparison, data on other children would have been useful, but such data were not systematically available in registers.

## Strength and limitations

Our study is a hospital-based review of prospectively recruited patients and hence is subject to biases. Firstly, our sample included selected patients from a referral institution with the potential to find more symptomatic lesions. Secondly, patients referred for echocardiography may be those with more severe patterns of lesions as patients with more severe lesions are more likely to seek medical attention. Thirdly, this was a retrospective review of echocardiographic reports performed by different operators; therefore there may have been some inter-rater variability not captured and conveniently addressed. This variability however, is likely to have been attenuated by the limited number of operators (two) and the fact that they have been collaborating for more than a decade and discussing difficult or uncertain cases together. Inherent to the retrospective nature of the study is the fact that earlier studies may not have used the guideline to conclude on the presence RHD. Although strict WHF criteria were used to characterize RHD pattern of valve lesion, some concerns remain about the significance and natural history of some minor valvular changes detected by echocardiography and only a long term follow up of these patients would help characterizing the true valve disease. Finally, we did not collect data on the studies which were normal. Despite of all the above shortcomings, our study is one of the few contemporary studies of prevalence and pattern of rheumatic valvular heart disease as seen on echocardiography in SSA children.

## Conclusion

In conclusion, RHD detected by echocardiography represents a sizable proportion of echocardiographies in the pediatric population in this largest referral echocardiography unit with frequent severe valvular lesions. Our findings add to the available evidence that Sub-Saharan Africa is the hot spot for RHD and further supporting that an A.S.A.P (Awareness, Surveillance, Advocacy Prevention) program, as already suggested by the Drakensberg declaration few years ago [22] and recently re-emphasized in the Mosi-o-Tunya Call to Action [23], should urgently be implemented in our setting.

## Abbreviations

ACC: American College of Cardiology
AHA: American Heart Association
RHD: rheumatic heart disease
SSA: Sub-Saharan Africa
WHF: World Heart Federation

## References

[1] Carapetis JR, McDonald M, Wilson NJ (2005). Acute rheumatic fever. Lancet.

[2] Carapetis JR, Steer AC, Mulholland EK, Weber M (2005). The global burden of group A streptococcal diseases. Lancet Infect Dis.

[3] Marijon E, Mirabel M, Celermajer DS, Jouven X (2012). Rheumatic heart disease. Lancet.

[4] Essop MR, Nkomo VT (2005). Rheumatic and nonrheumatic valvular heart disease: epidemiology, management, and prevention in Africa. Circulation.

[5] Marijon E, Ou P, Celermajer DS, Ferreira B, Mocumbi AO, Jani D, Paquet C, Jacob S, Sidi D, Jouven X (2007). Prevalence of rheumatic heart disease detected by echocardiographic screening. N Engl J Med.

[6] Beaton A, Okello E, Lwabi P, Mondo C, McCarter R, Sable C (2012). Echocardiography screening for rheumatic heart disease in Ugandan schoolchildren. Circulation.

[7] Grimaldi A, Ammirati E, Mirabel M, Marijon E (2013). Challenges of using ultrasounds for subclinical rheumatic heart disease screening. Int J Cardiol.

[8] Kane A, Mirabel M, Toure K, Perier MC, Fazaa S, Tafflet M, Karam N, Zourak I, Diagne D, Mbaye A (2013). Echocardiographic screening for rheumatic heart disease: age matters. Int J Cardiol.

[9] Remenyi B, Wilson N, Steer A, Ferreira B, Kado J, Kumar K, Lawrenson J, Maguire G, Marijon E, Mirabel M (2012). World Heart Federation criteria for echocardiographic diagnosis of rheumatic heart disease--an evidence-based guideline. Nat Rev Cardiol.

[10] Nishimura RA, Otto CM, Bonow RO, Carabello BA, Erwin 3rd JP, Guyton RA, O'Gara PT, Ruiz CE, Skubas NJ, Sorajja P, et al. 2014 AHA/ACC guideline for the management of patients with valvular heart disease: executive summary: a report of the American College of Cardiology/American Heart Association Task Force on Practice Guidelines. Circulation. 2014;129(23):2440–92. 10.1161/CIR.0000000000000029 24589852

[11] Damasceno A, Mayosi BM, Sani M, Ogah OS, Mondo C, Ojji D, Dzudie A, Kouam CK, Suliman A, Schrueder N (2012). The causes, treatment, and outcome of acute heart failure in 1006 Africans from 9 countries. Arch Intern Med.

[12] Grover A, Dhawan A, Iyengar SD, Anand IS, Wahi PL, Ganguly NK (1993). Epidemiology of rheumatic fever and rheumatic heart disease in a rural community in northern India. Bull World Health Organ.

[13] Padmavati S (1995). Present status of rheumatic fever and rheumatic heart disease in India. Indian Heart J.

[14] Avasthi G, Singh D, Singh C, Aggarwal SP, Bidwai PS, Avasthi R (1987). “Prevalence survey of rheumatic fever (RF) and rheumatic heart disease (Rhd) in urban & rural school children in Ludhiana”. Indian Heart J.

[15] Patel DC, Patel NI, Patel JD, Patel SD (1986). Rheumatic fever and rheumatic heart disease in school children of Anand. J Assoc Physicians India.

[16] Periwal KL, Gupta BK, Panwar RB, Khatri PC, Raja S, Gupta R (2006). Prevalence of rheumatic heart disease in school children in Bikaner: an echocardiographic study. J Assoc Physicians India.

[17] Rama Kumari N, Bhaskara Raju I, Patnaik AN, Barik R, Singh A, Pushpanjali A, Laxmi V, Satya Ramakrishna L (2013). Prevalence of rheumatic and congenital heart disease in school children of Andhra Pradesh, South India. J Cardiovasc Dis Res.

[18] Iung B, Vahanian A (2011). Epidemiology of valvular heart disease in the adult. Nat Rev Cardiol.

[19] Reeves BM, Kado J, Brook M (2011). High prevalence of rheumatic heart disease in Fiji detected by echocardiography screening. J Paediatr Child Health.

[20] Bhaya M, Panwar S, Beniwal R, Panwar RB (2010). High prevalence of rheumatic heart disease detected by echocardiography in school children. Echocardiography.

[21] Stevenson JG (1989). Two-dimensional color Doppler estimation of the severity of atrioventricular valve regurgitation: important effects of instrument gain setting, pulse repetition frequency, and carrier frequency. J Am Soc Echocardiogr.

[22] Mayosi B, Robertson K, Volmink J, Adebo W, Akinyore K, Amoah A, Bannerman C, Biesman-Simons S, Carapetis J, Cilliers A (2006). The Drakensberg declaration on the control of rheumatic fever and rheumatic heart disease in Africa. S Afr Med J.

[23] Mayosi BM, Gamra H, Dangou JM, Kasonde J, 2nd All-Africa Workshop on Rheumatic Fever and Rheumatic Heart Disease participants (2014). Rheumatic heart disease in Africa: the Mosi-o-Tunya call to action. Lancet Global Health.

